# BiaData Integration in the time of COVID: Getting to Yes with Enterprise-Wide Data Governance.

**DOI:** 10.23889/ijpds.v7i3.1802

**Published:** 2022-08-25

**Authors:** Amy Hawn Nelson

**Affiliations:** 1 Actionable Intelligence for Social Policy (AISP), University of Pennsylvania School of Social Policy & Practice

## Objectives

This presentation describes a successful framework for developing and implementing processes to support intradepartmental data access, integration, and use in the US. This case study describes on-going streams of work, 2019-present, to implement a new legal framework and data governance approach across a large health and human service agency.

## Approach

This work is conducted in partnership with a state agency and university-based researchers. The case study relies upon participatory action research as the primary approach, using mixed methods to inform inquiry, including interviews and deliberative dialogue to support action. An initial Data Landscape Overview was conducted from 2019-2020 through on-going meetings, document review, a survey of legal agreements, weekly calls with the Department’s Data Office, and structured interviews with 44 individuals. Deliberative dialogue has informed on-going action steps from 2020 to today as we have co-created a data governance roadmap, specifically focused on data governance and a new legal framework.

## Results

This work was performed in the context of a data strategy across five pillars: data governance & legal framework; workforce development & data literacy, data quality, data infrastructure, and data use. We focused on the first pillar—governance and legal frameworks. Drawing upon departmental exemplars and through deliberative dialogue with legal counsel, we built a new legal framework and successfully executed a three-tier legal approach using an Intradepartmental Memorandum of Understanding, Data Sharing Agreement, and Data Use Agreement. To date, we have co-created a department wide Data Sharing Guidebook, which includes a range of data pathways and data request processes; processes for developing high value data asset inventories; parameters for legal use; and clearly defined department-wide data governance—all operationalized within the established legal framework.

## Conclusion

Our work provides a model for large agencies to collaboratively develop and implement data governance (even during a pandemic). We are grounded in the understanding that data sharing is hard, and more relational than technical. Our goal is long-term sustainability, and therefore people have been central through all work streams.

